# Effect of the Direct Renin Inhibitor Aliskiren on Urinary Albumin Excretion in Spontaneous Type 2 Diabetic KK-*A* ^*y*^ Mouse

**DOI:** 10.1155/2013/519130

**Published:** 2013-06-02

**Authors:** Masako Furukawa, Tomohito Gohda, Shinji Hagiwara, Mitsuo Tanimoto, Satoshi Horikoshi, Kazuhiko Funabiki, Yasuhiko Tomino

**Affiliations:** Division of Nephrology, Department of Internal Medicine, Juntendo University Faculty of Medicine, 2-1-1 Hongo, Bunkyo-ku, Tokyo 113-8421, Japan

## Abstract

*Objective*. Although angiotensin II-mediated inflammation and extracellular matrix accumulation are considered to be associated with the progression of diabetic nephropathy, these processes have not yet been sufficiently clarified. The objective of this study was to determine whether the correction of the abnormal renal expression of MMPs and its inhibitors (MMPs/TIMPs) and cytokines following the administration of aliskiren to KK-*A* ^*y*^ mice results in a renoprotective effect. *Methods*. KK-*A* ^*y*^ mice were divided into two groups, that is, untreated (saline) and treated (aliskiren) groups. Systolic BP, HbA1c levels, and the albumin-creatinine ratio (ACR) were measured. The renal expression of MMPs/TIMPs, fibronectin, type IV collagen, MCP-1, and (pro)renin receptor ((P)RR) was examined using real-time PCR and/or immunohistochemical staining. Renal MAPK and NF-**κ**B activity were also examined by Western blot analyses and ELISA, respectively. *Results*. Significant decreases in systolic BP and ACR levels were observed in treated KK-*A* ^*y*^ mice compared with the findings in untreated KK-*A* ^*y*^ mice. Furthermore, increases in MMPs/TIMPs, fibronectin, type IV collagen, MCP-1, and (P)RR expression, in addition to MAPK and NF-**κ**B activity, were significantly attenuated by aliskiren administration. *Conclusions*. It appears that aliskiren improves albuminuria and renal fibrosis by regulating inflammation and the alteration of collagen synthesis and degradation.

## 1. Introduction

Recent studies suggest that chronic inflammation and extracellular matrix (ECM) accumulation promote the progression of diabetic nephropathy (DN) [1, 2]. We have also reported the increased renal expression of monocyte chemotactic protein (MCP)-1, fibronectin, and type IV collagen in KK-*A* ^*y*^ mice [3–5], a frequently used animal model of type 2 diabetes (T2D) [6]. Furthermore, angiotensin (Ang) II induces the phosphorylation of mitogen-activated protein kinase (MAPK) and increases nuclear factor (NF)-*κ*B binding activity in this mouse model [5]. Several studies have suggested that the renin-angiotensin system (RAS) is one of the major mediators of the progression of glomerular hypertension, inflammation, and tubulointerstitial fibrosis, which leads to the progression of DN [7–9].

Aliskiren is the first agent in a new class of orally effective direct renin inhibitors approved for hypertension treatment [10, 11]. In contrast to conventional RAS blockers, angiotensin-converting enzyme (ACE) inhibitors and Ang II type 1 receptor blockers (ARBs), aliskiren blocks RAS by directly inhibiting plasma renin activity and preventing the formation of both Ang I and Ang II, as demonstrated by basic and clinical findings [10, 12]. Data from the AVOID trial suggest that the addition of aliskiren to an ARB provides an additive antiproteinuric effect compared to that of the ARB alone [13]. From the ALTITUDE study [14], the potential cardiorenal benefit and safety of aliskiren in a broad range of high-risk patients with T2D remain controversial. Further basic studies will be required to understand the mechanism of action of aliskiren in the prevention of renal disease progression.

A strong correlation exists between interstitial expansion and glomerulosclerosis via alterations in renal blood flow, altered hemodynamics, and the direct effect of glucose; all of which promote the accumulation of ECM and the activation of profibrogenic and inflammatory cytokines [15]. Imbalances between the synthesis and degradation of glomerular ECM proteins by MMPs and its inhibitors (MMPs/TIMPs) are believed to play important roles in the progression of glomerular sclerosis in DN because high glucose or Ang II may induce alterations in the MMPs/TIMPs balance [16]. There is a positive crosstalk between inflammation and ECM synthesis that ultimately leads to chronic renal failure [17]. However, the role of MMPs/TIMPs within the context of diabetes remains controversial.

In the present study, we hypothesized that the direct renin inhibitor aliskiren may improve early DN via the attenuation of inflammatory cytokine expression and/or modulation of alteration MMPs/TIMPs expression in the kidneys of KK-*A* ^*y*^ mice.

## 2. Methods

### 2.1. Experimental Animals and Protocols

Six-week-old male KK/Ta Jcl and diabetic KK-*A* ^*y*^/Ta Jcl mice were purchased from CLEA Japan (Tokyo, Japan). The mice were individually housed in plastic cages with free access to food (rodent pellet diet NMF; 348 kcal/100 g, containing 5.5% crude fat) and water throughout the experiments. All mice were maintained in the same room under conventional conditions with a regular 12-h light/dark cycle and temperature controlled at 24 ± 1°C. All experiments were performed according to the guidelines of the Animal Care Committee of Juntendo University. Aliskiren was kindly given by Novartis Pharma AG (Basel, Switzerland). KK-*A* ^*y*^ mice were divided into two groups: (1) vehicle group (nontreatment) and (2) aliskiren (25 mg/kg per day) group (*n* = 10 or 11 per group). Aliskiren was subcutaneously infused via an ALZET micro-osmotic pump (Durect Co., Cupertino, CA, USA). Drug treatment was performed for 4 weeks (from 8 to 12 weeks of age). The drug doses were determined from the previous studies [18]. ALZET micro-osmotic pumps loaded with saline were used in the nontreatment KK-*A* ^*y*^ and KK mice groups. Age-matched untreated KK mice with nearly normal glucose tolerance levels were used as a control for the KK-*A* ^*y*^ mice. The experimental procedure was terminated when the mice reached 12 weeks of age. The mice groups were as follows: 8 weeks untreated KK mice group (Group1), 12 weeks untreated KK mice group (Group2), 8 weeks untreated KK-*A* ^*y*^ mice group (Group3), and 12 weeks untreated KK-*A* ^*y*^ mice group (Group4), and 12 weeks treated KK-*A* ^*y*^ mice group (Group5).

### 2.2. Biochemical Measurements

Body weight (BW), systolic blood pressure (SBP), fasting blood glucose (FBG) levels, hemoglobin A1c (HbA1c) levels, and the urinary albumin-creatinine ratio (ACR) were measured at 8 or 12 weeks of age. Urinary samples were collected for 24 h using a metabolic cage (mouse metabolic cage, CLEA Japan). Urinary albumin and creatinine levels were measured by immunoassays (DCA 2000 System; Bayer Diagnostics, Elkhart, IN). Glucose levels of blood obtained from the retro-orbital sinus were measured using a Glucocard meter (Kyoto Daiichi Kagaku, Kyoto, Japan). HbA1c levels were also measured by an immunoassay (DCA 2000 system). Blood pressure was measured by a pulse transducer system (Softron BP-98A, Tokyo, Japan). Standard deviations (SDs) of less than 5.0 were used to define the levels of blood pressure, as described previously [4, 19].

### 2.3. Real-Time PCR for MMP-2, MMP-9, TIMP-1, TIMP-2, Fibronectin, Type IV Collagen, MCP-1, and (Pro) Renin Receptor Expression

RNA was extracted from snap-frozen renal cortices using the RNeasy Mini Kit (Qiagen KK, Tokyo, Japan). RNA was reverse-transcribed using random decamer primers (Ambion, Austin, TX, USA) and MMLV Reverse Transcriptase (Life Technologies, Carlsbad, CA, USA). TaqMan real-time PCR was performed and analyzed according to the manufacturer's instructions (Applied Biosystems, Foster City, CA, USA). To measure gene expression in each tissue fraction, real-time PCR was performed using primers supplied with the commercially available assays obtained from Applied Biosystems (MMP-2: Mm01253624 _m1, MMP-9: Mm00600163 _m1, TIMP-1: Mm01341361 _m1, TIMP-2: Mm00441825 _m1, Fibronectin: Mm01256744 _m1, Type IV collagen: Mm01210125 _m1, MCP-1: Mm00441242 _m1, (Pro) renin receptor ((P)RR): Mm00510396 _m1, and Glyceraldehyde 3-phosphate dehydrogenase (GAPDH): Mm99999915 _g1). Each measurement was repeated four times. The relative mRNA level in the sample was normalized for GAPDH content.

### 2.4. Immunohistochemical Staining of MMP-2, MMP-9, TIMP-1, TIMP-2, and F4/80

The mice were killed at 8 or 12 weeks of age. Immunohistochemistry was performed with cryostat kidney sections (3 *μ*m) as described previously [4]. Cryostat kidney sections (3 *μ*m) were air-dried for 10 min and then fixed in cold acetone for 10 min. Nonspecific staining was blocked by incubation with avidin for 20 min and then with biotin for 20 min using the avidin-biotin blocking kit (Vector Laboratories, Inc., Burlingame, CA, USA). Endogenous peroxidase activity was inhibited by incubation with methanol containing 3% H_2_O_2_ for 10 min. The sections were then incubated with primary antibodies (Abs) in 20% normal goat or rabbit serum in 2% bovine serum albumin at 4°C overnight. The primary antibodies (Abs) were as follows: goat polyclonal anti-MMP-2 Ab (R&D Systems, Inc., Minneapolis, MN, USA), goat polyclonal anti-MMP-9 Ab (R&D Systems, Inc.), rabbit polyclonal anti-TIMP-1 Ab (Abbiotec, LLC, San Diego, CA, USA), rabbit polyclonal anti-TIMP-2 Ab (Abbiotec, LLC), and rat monoclonal anti-F4/80 Ab (MAC497GA; Serotec, Oxford, UK). The sections were incubated with secondary antibody. The secondary Abs were as follows: an anti-goat IgG (Dako, Carpinteria, CA, USA), anti-rabbit IgG (Vector Laboratories, Inc.), or anti-rat IgG (Cosmo Bio Co., Ltd, Tokyo, Japan). The sections were incubated with peroxidase-conjugated streptavidin antibodies (Dako), and 3,3-diaminobenzidine was then added for 5 min after which the slides were counterstained with hematoxylin. The staining of at least 10 glomeruli from each mouse was quantified using the KS-400 version 4.0 image analysis system (KS-400; Carl Zeiss Vision, Munich, Germany). The threshold was calculated as follows: the sum of the medium value of optical density in each group/the numbers of group. The number of F4/80-positive cells was counted in 10 randomly selected fields (×200). Analyses were performed by two investigators in a blinded fashion [4, 20].

### 2.5. Western Blot Analysis of p-p38, p-ERK1/2, and p-SAPK/JNK Expression

Portions of renal cortices samples were homogenized in lysis buffer containing a complete protease inhibitor cocktail tablet (Roche Diagnostics, Mannheim, Germany), 1 mM NaF, and 1 mM sodium orthovanadate (Sigma-Aldrich, Louis, MO, USA) and centrifuged. Appropriate volumes of the supernatant (20 *μ*g/lane) were mixed with an equal volume of sample buffer (312.5 mmol/L Tris–HCl, pH 6.8, 10% SDS, 50% glycerol, 10% 2-mercaptoethanol, and 0.025% bromophenol blue). SDS-PAGE gel electrophoresis and western blot analysis were performed according to standard protocols and were visualized using enhanced chemiluminescence immunoblot detection kits (ECL prime, Amersham Biosciences, Buckinghamshire, UK). The primary antibodies used were as follows: total p38, phosphorylated p38, total Erk1/2, phosphorylated Erk1/2, total SAPK-JNK, and phosphorylated SAPK-JNK (1 : 1000, Cell Signaling Technology, Inc., Danvers, MA, USA). HRP-conjugated second antibodies (Jackson Immuno Research Laboratories, West Grove, PA, USA) were used in this study. The concentration was measured by a LAS-3000 image system (Fujifilm, Tokyo, Japan).

### 2.6. Measurement of NF-*κ*B Activation

Nuclear extracts were obtained from renal cortices using a Nuclear Extract Kit (Active Motif, Tokyo, Japan) as described previously [5]. NF-*κ*B activation was measured using an NF-*κ*B Transcription Factor Assay Kit (Active Motif) according to the manufacturer's recommendations. NF-*κ*B activation was measured in triplicate using a spectrophotometer (Molecular Devices Spectra Max 340PC, Sunnyvale, CA, USA) at OD450.

### 2.7. Statistical Analysis

Data were expressed as the mean ± SD. Statistical differences between means were determined using the Bonferroni *t*-test. A value of *P* < 0.05 was considered statistically significant.

## 3. Results

### 3.1. Biochemical Parameters

There were no significant differences in the baseline values of BW, SBP, FBG, HbA1c, and ACR between the vehicle- and aliskiren-treated KK-*A* ^*y*^ mice at 8 weeks of age. However, these parameters except SBP in the vehicle-treated KK-*A* ^*y*^ mice were much higher than those in the vehicle-treated KK mice (Table 1).

The results of the biochemical parameters of the mice at the end of the 4-week experimental protocol are shown in Table 1. BW, HbA1c levels, and ACRs in vehicle-treated KK-*A* ^*y*^ mice were much higher than those in vehicle-treated KK mice. However, FBG levels and SBP did not differ among vehicle-treated KK mice and vehicle-treated KK-*A* ^*y*^ mice. SBP in aliskiren-treated KK-*A* ^*y*^ mice were significantly lower than those in the vehicle-treated KK-*A* ^*y*^ mice, throughout the treatment. The ACRs in aliskiren-treated KK-*A* ^*y*^ mice were significantly lower than those in the vehicle-treated KK-*A* ^*y*^ mice; however there was no statistically significant change in BW, FBG levels, and HbA1c levels between vehicle- and aliskiren-treated KK-*A* ^*y*^ mice.

### 3.2. Real-Time PCR Analysis of MMP-2, MMP-9, TIMP-1, TIMP-2, Fibronectin, Type IV Collagen, MCP-1, and (P)RR Expression in the Kidneys

**Figure 1:**
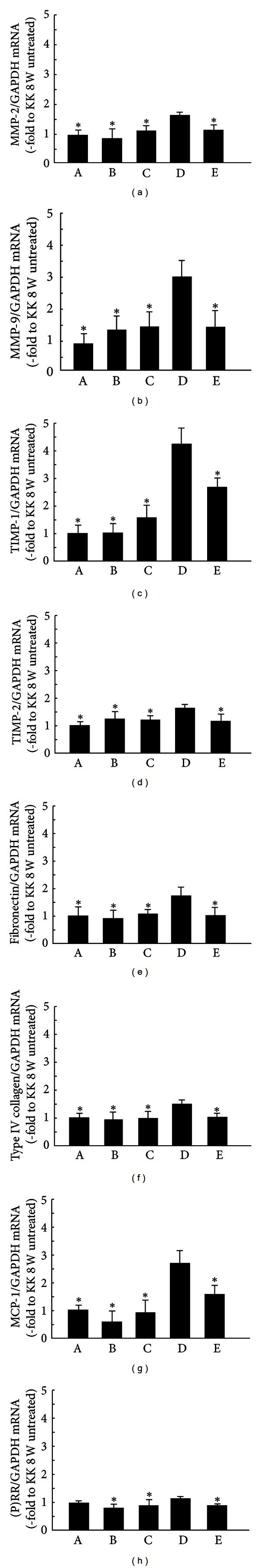
Expression of MMP-2, MMP-9, TIMP-1, TIMP-2, fibronectin, type IV collagen, MCP-1, and (P)RR in the kidneys of each mouse using real-time PCR (A: untreated KK mice at 8 weeks of age, B: untreated KK mice at 12 weeks of age, C: untreated KK-*A* ^*y*^ mice at 8 weeks of age, D: untreated KK-*A* ^*y*^ mice at 12 weeks of age, and E: treated KK-*A* ^*y*^ mice at 12 weeks of age). The ratio of MMP-2 (a), MMP-9 (b), TIMP-1 (c), TIMP-2 (d), fibronectin (e), type IV collagen (f), MCP-1 (g), and (P)RR (h) mRNAs to GAPDH mRNA is shown in the kidneys of mice from each group. These expressions were increased significantly in group 4 compared with groups 1, 2, and 3. This increase was attenuated in group 5 (*P < 0.001 versus untreated KK-*A* ^*y*^ mice at 12 weeks of age).

MMP-2, MMP-9, TIMP-1, TIMP-2, MCP-1, fibronectin, type IV collagen, MCP-1, and (P)RR mRNA expression in renal cortex tissues was increased significantly in group 4 compared with that in groups 1, 2, and 3 (*P* < 0.001; Figures 1(a)–1(h)). Aliskiren treatment (group 5) attenuated these increases in mRNA expression, resulting in an expression similar to that in the control KK mice (group 2) (*P* < 0.001; Figures 1(a)–1(h)).

### 3.3. Immunohistochemical Analysis of MMP-2, MMP-9, TIMP-1, TIMP-2, and F4/80 Expression in the Kidneys

**Figure 2:**
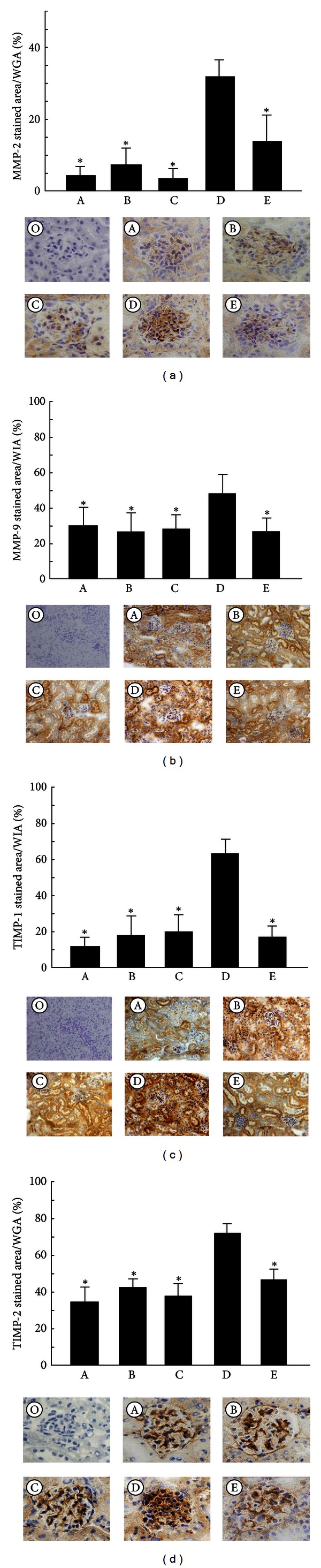
Immunohistochemical staining of MMP-2, MMP-9, TIMP-1, and TIMP-2 in the renal sections of each mouse (O: untreated KK-*A* ^*y*^ mice at 12 weeks of age without primary Ab, A: untreated KK mice at 8 weeks of age, B: untreated KK mice at 12 weeks of age, C: untreated KK-*A* ^*y*^ mice at 8 weeks of age, D: untreated KK-*A* ^*y*^ mice at 12 weeks of age, and E: treated KK-*A* ^*y*^ mice at 12 weeks of age). Stainings showing the expression of MMP-2 (a), MMP-9 (b), TIMP-1 (c), and TIMP-2 (d) in the kidneys of mice from each group. These expressions were increased significantly in group 4 compared with groups 1, 2, and 3. This increase was attenuated in group 5 (*P < 0.001 versus untreated KK-*A* ^*y*^ mice at 12 weeks of age). Images were taken at 400-fold (a and d) and 200-fold (b and c) magnification. WGA indicates whole glomerular area. WIA indicates whole interstitial area.

**Figure 3:**
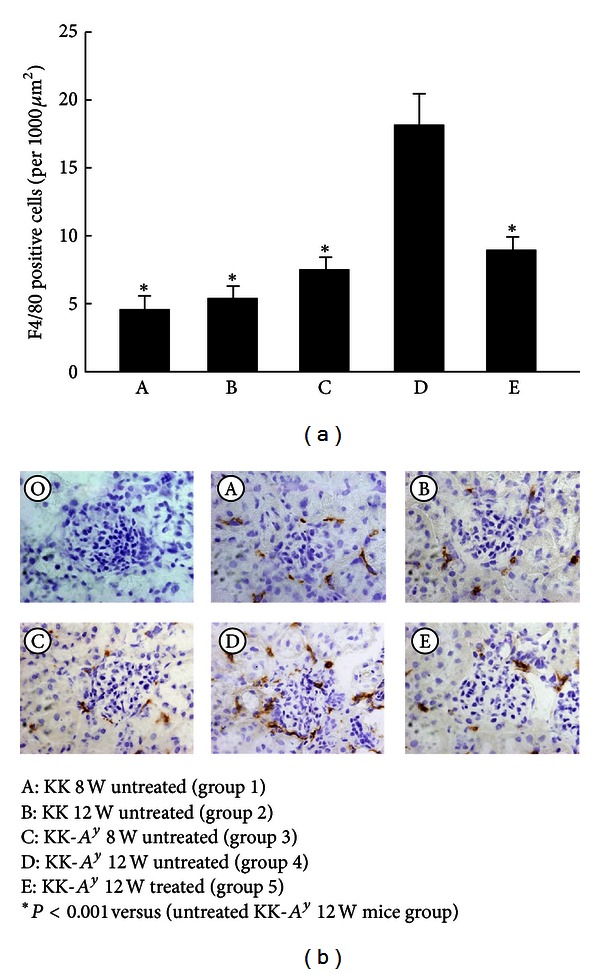
Number of F4/80 positive cells and staining of the renal sections of each mouse (O: untreated KK-*A* ^*y*^ mice at 12 weeks of age without primary Ab, A: untreated KK mice at 8 weeks of age, B: untreated KK mice at 12 weeks of age, C: untreated KK-*A* ^*y*^ mice at 8 weeks of age, D: untreated KK-*A* ^*y*^ mice at 12 weeks of age, and E: treated KK-*A* ^*y*^ mice at 12 weeks of age). The number of F4/80-positive cells was counted to determine the macrophage infiltration in each group. Macrophage infiltration increased significantly in group 4 as compared with groups 1, 2, and 3. This increase was attenuated in group 5 (*P < 0.001 versus untreated KK-*A* ^*y*^ mice at 12 weeks of age). Images were taken at 200-fold magnification.

MMP-2 (Figure 2(a)) and TIMP-2 (Figure 2(d)) expression was observed in glomeruli, especially within the mesangial areas. MMP-9 (Figure 2(b)) and TIMP-1 (Figure 2(c)) expression was localized in the proximal tubules. MMP-2, MMP-9, TIMP-1, and TIMP-2 protein accumulation was increased significantly in group 4 compared with that in groups 1, 2, and 3 (*P* < 0.001; Figures 2(a)–2(d)). Their expression was significantly suppressed in the aliskiren treatment groups (*P* < 0.001; Figures 2(a)–2(d)). F4/80-positive cells were localized in the proximal tubules. The number of F4/80-positive cells per 1000 *μ*m^2^ was significantly higher in group 4 than in groups 1, 2, and 3 (*P* < 0.001; Figure 3). These numbers were significantly lower in the aliskiren treatment groups (*P* < 0.001; Figure 3).

### 3.4. Western Blot Analysis of p-p38, p-ERK1/2, and p-SAPK/JNK in the Kidneys

**Figure 4:**
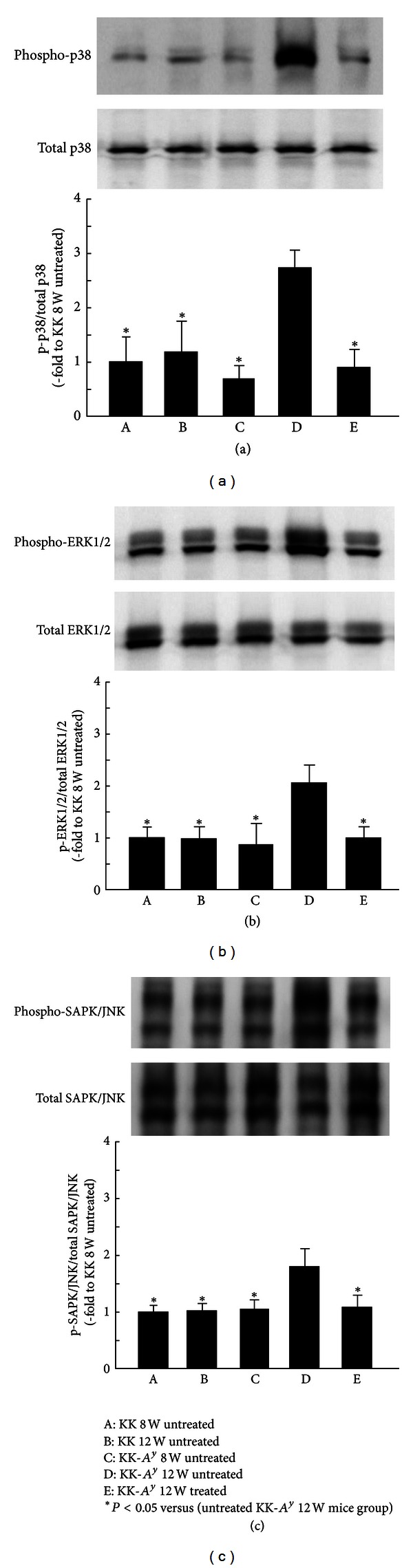
Mean protein levels of p-p38, p-ERK1/2, and p-SAPK/JNK in each mouse using Western blot analysis (A: untreated KK mice at 8 weeks of age, B: untreated KK mice at 12 weeks of age, C: untreated KK-*A* ^*y*^ mice at 8 weeks of age, D: untreated KK-*A* ^*y*^ mice at 12 weeks of age, and E: treated KK-*A* ^*y*^ mice at 12 weeks of age). The expression of p-p38 (a), p-ERK1/2 (b), and p-SAPK/JNK (c) proteins is shown in the kidneys of mice from each group. These expressions were increased significantly in group 4 compared with groups 1, 2, and 3. This increase was attenuated in group 5 (*P < 0.05 versus untreated KK-*A* ^*y*^ mice at 12 weeks of age).

To examine the effect of aliskiren on MAPK activity in the kidneys of KK-*A* ^*y*^ mice, Western blot analysis was performed (Figures 4(a)–4(c)). The protein expression of p-p38, p-ERK1/2, and p-SAPK/JNK in group 4 was significantly higher than that in groups 1, 2, and 3 (*P* < 0.05; Figures 4(a)–4(c)). Their expressions were significantly suppressed in the aliskiren treatment groups (*P* < 0.05; Figures 4(a)–4(c)).

### 3.5. Analysis of NF-*κ*B Activation in the Kidneys

**Figure 5:**
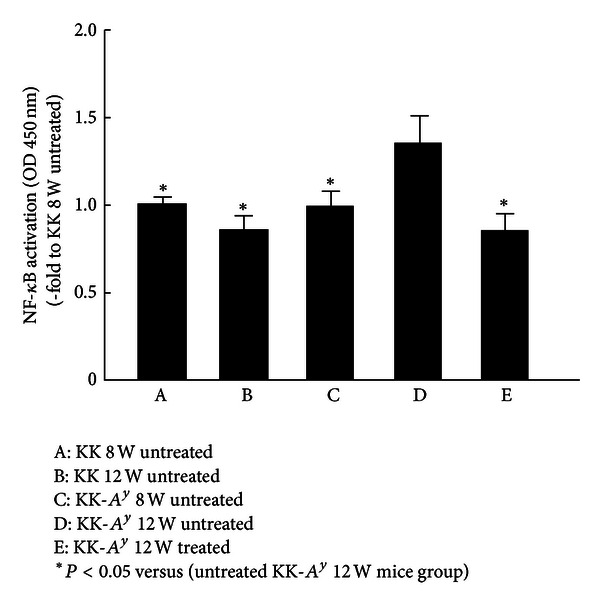
NF-*κ*B activation in each mouse (A: untreated KK mice at 8 weeks of age, B: untreated KK mice at 12 weeks of age, C: untreated KK-*A* ^*y*^ mice at 8 weeks of age, D: untreated KK-*A* ^*y*^ mice at 12 weeks of age, and E: treated KK-*A* ^*y*^ mice at 12 weeks of age). The nuclear extracts were prepared and processed for the analysis of NF-*κ*B activation. The levels of NF-*κ*B activation were measured at OD450. These expressions were increased significantly in group 4 compared with groups 1, 2, and 3. This increase was attenuated in group 5 (*P < 0.05 versus untreated KK-*A* ^*y*^ mice at 12 weeks of age).

NF-*κ*B activity in group 4 was significantly higher than that in groups 1, 2, and 3 as shown in Figure 5 (*P* < 0.05; Figure 5), and aliskiren suppressed this activation of NF-*κ*B (*P* < 0.05; Figure 5).

## 4. Discussion

The present study demonstrates for the first time that aliskiren dramatically ameliorated the levels of urinary ACR and renal fibrosis by improving inflammation and the alteration of MMPs and/or TIMPs expression in T2D KK-*A* ^*y*^ mice.

It is well known that the RAS pathway is activated in the diabetic kidney. Ang II increases the levels of MCP-1, which induces monocyte immigration and differentiation to macrophages and then augments ECM production and tubulointerstitial fibrosis [3, 21]. We have already demonstrated the presence of macrophage infiltration and increased MCP-1 expression in KK-*A* ^*y*^ mice [3, 4]. Recently, Tesch [22] reported that MCP-1 may have significant diagnostic value in evaluating the renal inflammatory response in DN. Moreover, Ang II induces the accumulation of type IV collagen and fibronectin through an imbalance within the MMPs/TIMPs system, which reduces the degradation of matrix proteins [16, 23]. However, the crosstalk between inflammation and the MMPs/TIMPs system remains controversial. In this study, we focused on MMP-2, MMP-9, TIMP-1, and TIMP-2 because these are the main regulators of the metabolism of type IV collagen, the most important ECM protein in DN, although MMP-2 also degrades fibronectin [24].

Data suggesting a link between MMPs/TIMPs dysregulation and DN also exist but are contradictory. Rodent models of diabetes revealed the decreased expression of MMP-2 [25–27] and MMP-9 [28, 29] in renal tissues. By contrast, the expression of the MMP inhibitors TIMP-1 [30, 31] and TIMP-2 [16] was increased. In vitro, both decreases and increases in MMP-2 [32, 33] and MMP-9 [32, 34] secretion have been demonstrated when rodent mesangial and podocyte cells were cultured under high glucose conditions. In contrast, TIMP-1 [35] activity was increased in mesangial cells under high glucose conditions. In patients with DN, both serum and urine MMP-2, MMP-9, and TIMP-1 concentrations increased with worsening glomerular lesions [36–39]. Several possible explanations can be offered to reconcile these differences: (1) differences in animal models, (2) differences in environment factors (i.e., in vitro and in vivo), and (3) differences in the stage of DN (early or late stage of kidney disease). The present study demonstrated for the first time that both the mRNA expression of MMP-2, MMP-9, TIMP-1, and TIMP-2, and the expression of those proteins clearly increased in the kidneys of T2D mice. Interestingly, MMP-2 and TIMP-2 expression was observed in the glomeruli, and their expression was more prominent in the mesangial areas than in the proximal tubules. MMP-2 and TIMP-2 may have been primarily related to glomerular basement membrane thickening and mesangial matrix expansion in this experiment. There is a possibility that at least some of the effects of MMP-9 and TIMP-1 could be mediated by MCP-1 system modulation, as macrophage infiltration was mainly observed in the proximal tubules. In contrast, the effects of MMP-2 and TIMP-2 might be related to Ang II and dependent on renin without dependence on inflammatory mediators such as MCP-1 and macrophages. Although we also sought to measure the mRNA or protein levels of IL-6 in glomeruli and urine, respectively, its levels were too subtle to be detected accurately. However, MCP-1 was detectable in urine, and the levels of urinary MCP-1 tended to be lower after aliskiren treatment, although significant differences were not observed (data not shown). Therefore, we consider that MCP-1 might be the most important marker of the progression of DN in this stage.

Ang II upregulation is known to modify the activity of tyrosine kinases and phosphatases, activate MAPK, and stimulate transcriptional factors [40, 41]. In addition, renin induces direct (P)RR signaling, leading to MAPK activation that is independent of Ang II [42–44]. Feldt et al. [45] reported that plasma renin activity can be blocked by aliskiren administration; however, the direct (P)RR signaling resulting in MAPK activation and (P)RR gene expression cannot be inhibited by aliskiren. In contrast, Feldman et al. [46] indicated that aliskiren inhibits (P)RR gene expression in vivo. Thus, we sought to determine whether aliskiren can alter (P)RR gene expression in our mouse model. These beneficial effects of aliskiren might be related to the suppression of renal MAPK activity, which may be Ang II- and/or (P)RR-dependent. We speculate that in addition to lowering systemic BP and blocking the circulating and tissue RAS system, aliskiren may have an antifibrotic action that may be mediated by (P)RR as follows: suppression of (P)RR gene expression may decrease the numbers of receptors, dampen intracellular fibrotic pathways induced by (pro) renin, and negate the gain in activity of receptor-bound renin. Because our data could not indicate whether aliskiren inhibits Ang II or (P)RR pathways, more work will be needed to clarify this issue.

Several possible explanations can be offered to reconcile these differences of the results between the ALTITUDE study and the present study. (1) It is certainly difficult to compare simply the results of human model with those of mouse model. In our mouse model, the levels of serum creatinine did not increase, although the levels of urinary ACR were relatively high [4]. (2) Adverse events of hyperkalemia and hypotension which were observed in aliskiren monotherapy such as our study were probably much lower than those in combination therapy of aliskiren added to standard-of-care renin-angiotensin blockade such as ALTITUDE study. Therefore, we consider that there is the possible benefits of aliskiren monotherapy in early stage of diabetic patients. However, we need further study under various conditions to determine the possible benefits of aliskiren, because unlike the ALITITUDE study, the end point of our study was not end-stage renal disease or cardiovascular death.

The main limitations of our study are as follows. The treatment protocol used in this study affected blood pressure. Thus, the beneficial effects of aliskiren might be partly derived from antihypertensive effects. Furthermore, we did not demonstrate the natural course of renal MMPs/TIMPs, type IV collagen, fibronectin, and MCP-1 expression until the late stages of DN. However, the objective of this study was to determine the changes in cytokine and MMPs/TIMPs levels in response to high glucose or Ang II and to observe aliskiren-induced changes in their levels during the early stages of DN.

In conclusion, it appears that aliskiren reduces albuminuria and inhibits the renal fibrosis in diabetic kidney disease. These effects might be related to the regulation of the alteration between collagen synthesis and degradation and to inflammation via the activation of MAPK and NF-*κ*B via Ang II- and/or (P)RR-mediated actions.

## Figures and Tables

**Table 1:** Biochemical profiles of KK mice and KK-*A* ^*y*^ mice.

Mice *n*	Untreated KK 7	Untreated KK-*A* ^*y*^ 10	Treated KK-*A* ^*y*^ 11
8 weeks of age			
Body weight (g)	26.0 ± 0.3*	31.1 ± 0.8	31.3 ± 1.6
Systolic blood pressure (mmHg)	103 ± 4	104 ± 2	105 ± 2
Urinary albumin-creatinine ratio (mg/g·Cr)	47 ± 22*	158 ± 54	150 ± 78
Fasting blood glucose (mg/dL)	83 ± 5*	110 ± 6	110 ± 7
HbA1c (%)	4.0 ± 0.2**	4.3 ± 0.3	4.2 ± 0.1
12 weeks of age			
Body weight (g)	32.8 ± 0.5*	38.9 ± 0.8	38.8 ± 1.9
Systolic blood pressure (mmHg)	116 ± 5	116 ± 28	96 ± 2*
Urinary albumin-creatinine ratio (mg/g·Cr)	24 ± 10*	552 ± 268	112 ± 99*
Fasting blood glucose (mg/dL)	106 ± 5	108 ± 7	105 ± 10
HbA1c (%)	4.3 ± 0.2*	6.9 ± 0.4	6.7 ± 0.2

Data are expressed as means ± SD.

*P < 0.01 (versus untreated KK-*A* ^*y*^ mice group), **P < 0.05 (versus untreated KK-*A* ^*y*^ mice group).
